# Comment to “Very late intubation in COVID-19 patients: A forgotten prognosis factor?”

**DOI:** 10.1186/s13054-022-04033-w

**Published:** 2022-07-11

**Authors:** Ricard Mellado-Artigas, Luigi Zattera, Enric Barbeta, Carlos Ferrando

**Affiliations:** 1 Surgical ICU (Department of Anaesthesiology), Hospital ClínicInstitut D’investigació August Pi i Sunyer, Villarroel 170, 08025 Barcelona, Spain; 2 CIBER de Enfermedades Respiratorias, Instituto de Salud Carlos III, Madrid, Spain

Dear Editor,

We have read the manuscript published by Camous and colleagues in this journal [1]. We want to congratulate the authors for their effort in describing outcomes in ventilated COVID-19 patients and its relationship to the time of intubation. The authors showed that intubation after 7 days of dexamethasone start was associated with a dismal outcome, while intubation between days 1 and 7, as compared to intubation before day 1, was not linked to worse results.

The optimal timing for mechanical ventilation initiation, including COVID-19 patients, has been a matter of debate with studies supporting or refuting the effect of a delay in intubation on outcomes, and overall uncertain results [2, 3]. In the absence of clinical trials to provide high-quality evidence, observational studies are used to sustain daily practice. Unfortunately, observational studies often present with large imbalances in relevant variables between treatment groups that preclude an easy estimation of treatment effects. In other words, groups tend to differ at baseline in the degree of severity of their illness. Nonetheless, if confounding can be controlled for, these data might offer valuable information on a particular treatment effect.

In the present work, very late intubation was associated with higher mortality. While the potential role of a delay in intubation on outcome cannot be ruled out, we want to highlight that patients among groups largely differed in important variables: first, the early intubation group presented at baseline a median ROX index as low as 3, as well as higher illness severity, as reported by higher SOFA score, while the very late group displayed a median ROX of 6, making these patients populations not comparable in terms of both baseline severity and probability to be intubated. We suggest therefore an analysis after adjusting for these important covariates [4]. Second, the very late group received more frequently tocilizumab as an adjunctive therapy: although the authors hypothesized that this was due to a longer time between steroid treatment and intubation, intubation is not a formal contraindication for such treatment. Usually, patients receiving tocilizumab show a higher inflammatory burden despite steroid treatment which has been identified as marker of disease severity [5].

In conclusion, we think that the data presented in this brief report, although of great interest, might present important limitations as residual confounding could not be excluded.

## Data Availability

No applicable.
