# Supplementary material for: Comment to “Very late intubation in COVID-19 patients: A forgotten prognosis factor?”
Source: Crit Care. 2022 Jul 11;26:212. doi: 10.1186/s13054-022-04033-w (PMC9272866; doi:10.1186/s13054-022-04033-w)
Supplement: Related texts

# Reply: Authors’ response

**Authors:** Laurent Camous, Jean-David Pommier, Frederic Martino, Benoît Tressieres, Alexandre Demoule, Marc Valette

Dear Editor,

We thank *Mellado-Artigas* et al. for their interest in our work and for their suggestions. As these authors underline, timing and indications of mechanical ventilation of SARS-CoV-2 pneumonia are still debated. We tried to understand the high mortality of lately intubated COVID patients, after careful studies of the potential bias and not comparing strategies of care.

For *Mellado-Artigas* et al. groups of our study are not comparable because of the baseline differences in ROX and SOFA. However, the description of our groups was based on the day of mechanical ventilation. As ROX scores at intubation were not different in between the three groups of intubated patients, we believe that respiratory condition at intubation was similar. Another potential bias discussed by *Mellado-Artigas* et al. was the difference in-between groups in the use of tocilizumab. However, as there are no robust data on mortality, incidence of nosocomial infection and ventilator-free days after tocilizumab treatment, we chose in our center not to use tocilizumab in mechanically ventilated patients in view of the potential infectious risk. There was no difference in inflammatory markers (CRP and D-dimer levels) at baseline between groups.

The impact on prognosis of late intubation in COVID patients has been discussed by others [2, 6]. The different hypotheses were discussed in our study: potential patient-self-induced lung injury (p-SILI) due to prolonged high flow nasal oxygen therapy or worsening of lung damages during steroid treatment.

Randomized trials are needed to confirm our data and to better understand the mechanisms of mortality in late intubated COVID patients. *Mellado-Artigas *et al. proposed the use of a propensity score-based cohort analysis as a tool to compare patients. We think that pairing of ARDS patients is difficult, as PaO_2_/FiO_2_ ratio or radiological abnormalities on CT-scan are rough markers of lung damage severity.
